# State-civil society partnerships for HIV/AIDS treatment and prevention in Ghana: exploring factors associated with successes and challenges

**DOI:** 10.1186/s12913-016-1598-9

**Published:** 2016-08-02

**Authors:** Martin Hushie, Cephas N. Omenyo, Jacob J. van den Berg, Michelle A. Lally

**Affiliations:** 1 Department of Behavioural Sciences, School of Allied Health Sciences, University for Development Studies, P.O. 1883, Tamale, N/R Ghana; 2 College of Education, University of Ghana, P.O. Box LG 1181, Legon, Accra Ghana; 3 Division of Infectious Diseases, The Miriam Hospital, 164 Summit Avenue, Providence, RI 02906 USA

**Keywords:** State-civil society partnerships, HIV/AIDS response, Ghana, Qualitative research

## Abstract

**Background:**

The past decade has seen an increased number of state-civil society partnerships in the global Human Immunodeficiency Virus/Acquired Immune Deficiency Syndrome (HIV/AIDS) response of many countries. However, there has been limited research carried out concerning the successes and challenges of these partnerships.

**Methods:**

In-depth qualitative interviews were conducted with 23 participants from 21 different state-civil society partnerships throughout Ghana including all three major geographical zones (Northern, Middle, and Southern zones) to examine the nature of these partnerships and their positive and negative effects in responding to the national HIV/AIDS epidemic.

**Results:**

Major themes included: 1) commitment by the government and civil society organizations to work cooperatively in order to support the development and implementation of HIV/AIDS interventions in Ghana; 2) the role of civil society organizations in facilitating community mobilization; capacity building; and information, resources and skills exchange to increase the efficiency and effectiveness of these partnerships for HIV prevention and treatment; and 3) significant challenges including funding issues and other structural barriers for these partnerships that need to be addressed moving forward.

**Conclusions:**

Future research should focus on examining the impact of recommended changes on state-civil partnerships and studying the extent and nature of these partnerships in other countries in order to establish the generalizability of the findings from this study.

**Electronic supplementary material:**

The online version of this article (doi:10.1186/s12913-016-1598-9) contains supplementary material, which is available to authorized users.

## Background

The last decade has seen an increasing proliferation of state-civil society partnerships for Human Immunodeficiency Virus/Acquired Immune Deficiency Syndrome (HIV/AIDS) prevention, treatment, care and support in at least 35 sub-Sahara African countries, which includes Ghana, Kenya, Uganda, Tanzania, Nigeria and Malawi [1, 2]. The growing movements toward greater coordination of aid, the strengthening of health care systems in the current funding environment, and efforts to scale up access to health interventions have all been cited as major factors contributing toward this proliferation [3–8].

These global change processes have tended to alter the balance between state and market to produce a system of organizational arrangements for the delivery of health care services. This is particularly striking in developing countries where Civil Society Organizations (CSOs) that include Non-Governmental Organizations (NGOs), Faith-Based Organizations (FBOs), and Community-Based Organizations (CBOs) now supplement, and in some cases have displaced the traditional role of the state in health care [9–13]. Thus, although it had historically been deemed the responsibility of the state to assure the provision of adequate health care to the wider population, national governments in developing countries, such as Ghana, are increasingly fostering collaborative arrangements with the non-profit sector in the national HIV/AIDS response.

CSOs, likewise, in their quest to expand the scope of their service delivery and advocacy activities, are progressively engaged in actions resulting in strategic alliances with the state in the fight against the HIV/AIDS epidemic. This signals change in the longstanding organizational system supporting the national HIV/AIDS response and in the form and functions of CSOs themselves, as they try to adjust to new roles and models for social services delivery. While these novel methods of organizing and delivering services have been unfolding across developing countries for a number of years, there has been limited research regarding their drivers, structure, successes and challenges [14–16]. Understanding the factors associated with successful partnerships is critical globally as countries struggle to fight the global HIV/AIDS pandemic with limited resources.

### The Ghana AIDS Commission

The Ghana AIDS Commission (GAC) was established in 2002 as the highest coordinating and decision-making body in the HIV/AIDS response in this country. Through external donor funding, the GAC has increasingly engaged CSOs including NGOs, FBOs and CBOs as co-implementers. As of 2009, the GAC has been working with approximately 30 large international and national CSOs, considered to have better organizational and technical capacity in terms of funding, management and program/project personnel that serve as intermediaries, providing funding and capacity building for smaller CSOs, believed to have limited funding and technical skills for implementing district-wide HIV prevention projects country-wide.

The challenge for the GAC is how to effectively harness the role of CSOs in the fight against the national HIV/AIDS epidemic amidst severe fiscal constraints [17]. However, an exploration of the relationships developing between the GAC and CSOs has not been done, and how their interactions enable or constrain the national HIV/AIDS response is poorly understood. There is minimal guidance on how the GAC can best work with civil society partners, and there is little sharing of lessons learned on the mechanisms and approaches currently being utilized [18]. This is in spite of the widely accepted view that civil society is a key sector in the national HIV/AIDS response, is often able to achieve results in areas inaccessible to government, and can be an important service provider [6]. Without the effective involvement of civil society, responses to HIV/AIDS are likely to be significantly compromised [19].

### Conceptualizing the state, CSOs and partnership

In order to analyze the partnership between the GAC as a state institution and CSOs, it is important to define three terms within the institutional context of the HIV/AIDS response in Ghana. What is meant by the terms "state," "civil society organization," and "partnership?"

For the purposes of this study, the following definitions of the terms are used to understand state-civil interactions in the national response to HIV/AIDS.

The term state can be used to describe government institutions whose aim is to create and maintain public order and deliver public goods and services. State organizations include the various levels of government: bureaucracies organized often as departments or ministries; state appointed bodies and agencies that provide public services at the national and sub-national or local levels [20]. In this research, the GAC is viewed as the major government institution, which CSOs have been working with since its inception. However, given the multi-sectorial nature of the national response, it was also conceptualized that CSOs by design might have some working relations with the Ghana Health Service (GHS) that the GAC engages with to address the national HIV/AIDS epidemic.

The term civil society organization is not understood in the same way globally. A useful definition of civil society that was adopted in the present study is "that sphere of social interaction between the household and the state, which is manifest in norms of community co-operation, structures of voluntary association, and networks of public communication" [21]. CSOs include people's movements, citizens' groups, religious institutions, women's organizations, and indigenous people's associations or grassroots organizations directly serving individuals of their community. NGO has become the most commonly used word to describe such organizations. The World Bank defines NGOs as "private organizations that pursue activities to relieve suffering, promote the interests of the poor, protect the environment, provide basic social services, or undertake community development" [22].

Finally, a review of the published research and theoretical literature on collaboration revealed that there is no single definition of a partnership; instead, a plethora of terms have been used. Some theorists focus on strategic alliances [23], action sets [24], joint ventures [25], or inter-organizational relations [26]. For this study, a partnership or collaboration was defined as "a process in which autonomous or semi-autonomous actors interact through formal and informal negotiation, jointly creating rules and structures governing their relationships, and ways to act or decide on the issues that brought them together; it is a process involving shared norms and mutually beneficial interactions" [27].

To the knowledge of the researchers, this study is the first of its kind to demonstrate the growing movement towards state collaboration with CSOs to achieve wider national health goals in the HIV/AIDS response. The aim of this study was to provide an investigation of the origins and drivers of such partnerships, the nature and processes of their collaboration, and the barriers and facilitators to achieving successful partnerships. The key research questions that were qualitatively explored are as follows: (1) how do state-CSO partnerships for HIV/AIDS emerge? (2) what are their distinguishing characteristic features? and (3) what successes or challenges do partners' perceive to be associated with partnerships?

## Methods

### Study Design

A qualitative methodology [28, 29], building on multiple interviews was adopted to allow for an in-depth analysis and detailed description of the origins and elements of the partnerships and what their successes and challenges might be in Ghana.

### Study setting

Ghana is located in West Africa. It borders Cote d'Ivoire to the west, Burkina Faso to the north, Togo to the east and the Gulf of Guinea to the south. It was the first African country south of the Sahara to gain independence from British colonial rule in 1957. After decades of military rule, Ghana made a transition to democratic rule in 1992 and remains one of the few functioning multi-party parliamentary democracies in the highly unstable West-African sub-region. Economically, it has built reputation as a stable liberal economy and was deemed to have attained a lower middle income country status in 2010.

### Sampling

In this study, a random sampling procedure that would require having a complete list of all the partnerships in the country from which samples could be drawn was not used because such a list does not presently exist. Hence, it was purposefully determined that identified organizational partnerships should at least be representative of all the three major geographical zones in the country ― the Northern, the Middle and Southern zones, which reflect Ghana's ethnic and cultural diversity and inequalities in the distribution and access to health care, with the Northern zone and rural areas being the most deprived regarding access to existing health resources [30]. Initial contacts were made with representatives of the partnerships in these three geographical zones that spanned both large CSOs and their sub-recipients (SRs) (i.e., smaller NGOs, FBOs and CBOs) to ensure that they had ever been or were currently engaged in collaborative relationships. Following an initial interview with two GAC participants of the partnerships, they were asked to nominate potential research participants across the three geographical zones. In all, 30 CSOs were contacted but only 21 participated. The remaining nine could not be included because the CSO representatives of the partnerships were not available at the time of the interviews. Informed consent was obtained as per applicable guidelines and as approved by site institutional review boards.

### Data collection

Two major methods were utilized and triangulated to gather data – semi-structured, in-depth qualitative interviews (Additional file 1) and review of organizational documents of the GAC and CSOs. The wider literature on CSOs and HIV/AIDS and information on the internet was used to enhance the reliability and external validity of the findings. In total, 23 semi-structured in-depth qualitative interviews were conducted with 21 participants from the CSOs and two from the GAC in 2012–2013. The interviews were conducted consecutively until a point of saturation (e.g., when additional interviews yielded no new insights for the study) was reached. In each case, interviews were held with the Program/Project Officer, Executive Director/Secretary or other person in a position to represent a range in the partnerships.

### Data analysis

Interviews were conducted in English, audio-recorded and transcribed verbatim. Interview transcripts were examined for accuracy and transcription errors were corrected. All participant identifiers were removed. Each transcript was coded manually by two independent coders to organize the resulting data. Codes were then compared and any discrepancies were resolved prior to final analysis. An interpretive approach that emphasizes constructing meaning within and across the interviews’ contexts of partnerships was used. This approach required that codes be developed to provide a basis for categorizing and analyzing the data along the key research questions [29]. The data analysis as a whole consisted of two stages: (1) developing summaries of each partnership (from interviews and documentation); describing how the partnership formed, when it occurred, and who was involved; and the nature and elements of the collaboration process and its major effects; (2) these descriptions were then used as a basis for more focused summaries in which the information was coded and reorganized around the themes central to this research – the emergence of the partnerships, their nature, successes and challenges.

## Results

### Findings from the partnerships

The reported increasing involvement of CSOs has led to profound changes in the institutional and governance arrangements for HIV/AIDS treatment, prevention, care and support in Ghana. The organizational partnerships studied are presented with respect to the conditions for their emergence, the contribution of the partners, how the partnerships are administered and their advantages and disadvantages. In order to ensure the anonymity and confidentiality of the participants and their organizations, they are identified only by their size (small or large) and the three geographical zones (Northern, Middle, or Southern) in which they are located.

### History and drivers of CSOs’ engagement

Evidence gathered from organizational documents and participants in this study revealed that the first major driver for the involvement of CSOs was the establishment of the GAC by the Parliamentary Act 613 in 2002 to coordinate the country’s HIV/AIDS response. Subsequently, CSOs’ engagement was to be shaped significantly by the availability of the Ghana AIDS Response Fund (GARFUND) of $28.7 million (2002–2005) from the World Bank to support HIV programs of the GAC. Among others, the fund aimed to: (i) support the GAC to implement a multi-sector response; (ii) expand the Ministry of Health’s role to engage CSOs; and (iii) finance eligible activities conducted by CSOs and state institutions to promote a rapid scaling-up of the HIV/AIDS response country-wide [31].

The GAC has since been working with CSOs, guided by specific implementation guidelines, and 5-year national HIV/AIDS strategic frameworks/plans with the wider objective of fighting the HIV/AIDS epidemic through treatment, prevention, care and support interventions [17, 32, 33]. The first National Strategic Framework (2001–2005) targeted prevention and care services to reduce high-risk behaviors and exposure to HIV, along with identifying and treating the disease [32]. During this period, the GAC worked with thousands of CSOs that had emerged to execute the national response. Although they were often contracted on an ad-hoc basis with small grants to do short-term HIV projects, the GAC was to find itself overstretched as new priorities of the disease emerged, such as deepening behavior change communication and the need to focus on prioritized areas as a means for controlling the epidemic.

In response to these new demands, the GAC had to reconsider how it worked with CSOs. This meant that CSOs had to be funded to implement projects through the development and submission of competitive project proposals in prioritized areas for review and selection by a Proposal Review and Appraisal Committee. From the GAC’s perspective, this would reduce the number of NGOs it had to coordinate and make its Monitoring and Evaluation (M&E) work more cost-effective [33].

Another purpose of this new strategy has been to channel funds through large CSOs with better organizational and technical capacity for subsequent disbursement to smaller CSOs, such as NGOs, FBOs and CBOs with limited organizational capacities. The large CSO recipients then become responsible for building the capacity of the small CSOs, monitoring and evaluating their activities, making sure their projects get completed and ultimately furnishing the GAC with standardized data about their work. In complementing this view, one of the large CSO participants in the Southern zone stated:

*"…You know, the GAC cannot move from their office to be doing HIV work in the districts and communities. So they work with people like us and other smaller NGOs to get their work done across Ghana…it will be a monitoring nightmare for GAC to try and work with all these small NGOs, trying to reach people…So we help them to achieve their objectives. Without organizations like ours, I do not think they can do their work."*

In recruiting small CSOs as co-implementers, the large CSOs use various criteria including their need to possess office space, ancillary staff, strong grassroots presence and linkages with existing government health facilities whose professional input will be required to conduct HIV Testing and Counseling (HTC) programs. Concerning this, one of the study participants from a small NGO in the Southern zone described the process by which small CSOs are recruited as follows:

*"Before you are taken on board as a SR, you need to provide certain documentation. So they came to do a critical assessment of our financial management system, the staff available, their capacity, years of experience, track record…And for us, we were looking to them being able to support us, give us the requisite technical assistance/support, the funding that we need, and basically, that is what we want."*

A small CBO participant from the Middle zone captured the nature of the funding relationship between the large and small CSOs and how this benefits the latter as follows:

*"Because they [large CSOs] are closer to the powers that pay, like the GAC, they can easily get funding. But for us, it is difficult as they do not deal directly with small CBOs. So it is good that we have relations with them.*

Subsequently, following the initial funding of the GAC by the World Bank, CSOs have been supported by multi-lateral and bilateral donor agencies such as: United States Agency for International Development (USAID); Department for International Development of the United Kingdom (DFID); Danish International Development Agency (DANIDA); and Global Fund to Fight AIDS, Tuberculosis and Malaria (Global Fund).

Most of the CSOs included in this study were already engaged in some sexual/reproductive health, HIV/AIDS and other health initiatives, but with limited funding from other international NGOs and charities abroad. Consequently, the funding opportunities provided by the GAC were viewed not only as a means by which they could expand their work to various vulnerable groups, such as youth, orphans, women, and people living with HIV (PLHIV) but also, a means by which they could tap into the knowledge and skills provided by GAC through its capacity building initiatives to enhance their work. On this point, one of the large NGO participants in the Northern zone indicated that:

*"We were already doing some programs in HIV/AIDS and sexual and reproductive health but we were doing that with small funding from different charities engaging in small initiatives in just some two or three communities across the region. So when we saw the advert, we realized that it was an opportunity for us to expand our services to vulnerable groups in our communities with the additional resources coming from GAC, and so we decided to put in an application. That is how come we became involved with the GAC."*

Thus, external donor support has been crucial for the engagement of CSOs, and highlights the key role of the GAC through which funds have been channeled to legitimatize the work of CSOs to implement the national HIV/AIDS response.

### Structure of current relationships between the GAC and CSOs

**Fig. 1:**
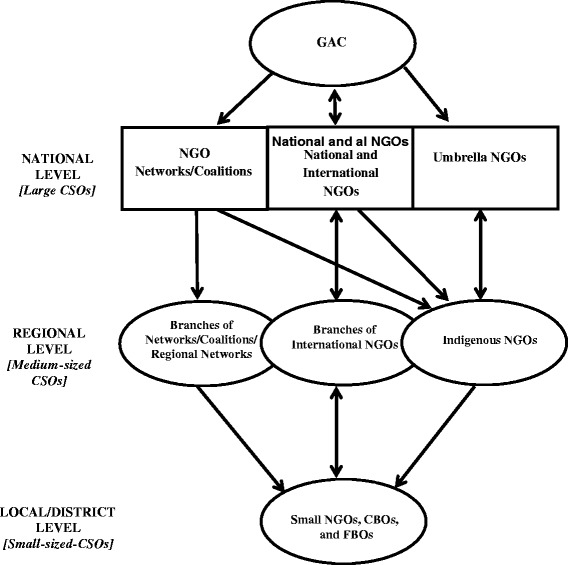
Current institutional and partnership arrangements between the GAC and CSOs in the HIV/AIDS response in Ghana

The evidence gathered for this study revealed that the GAC currently maintains a complex relationship with different types of CSOs at three major levels – national, regional and local/district in the response to HIV/AIDS (Fig. 1). At the national level, CSOs are involved mainly in national strategic planning and programming, and they include interest-based network/coalitions, such as the Ghana Business Coalition against HIV/AIDS and the Ghana Network of Persons Living with HIV/AIDS (NAP+); national and international NGOs; and umbrella NGOs, such as the Coalition of NGOs in Health, Ghana HIV/AIDS Network, and Christian Health Association of Ghana.

At the regional level, the CSOs include branches of national NGO networks/coalitions (interest groups) such as NAP+, and regionally-based interest groups of networks/coalitions; local NGOs that either constitutes branches of International NGOs (INGOs) registered as local organizations or indigenous organizations that have been established with assistance from INGOs. At this level, most of the NGOs are described as "medium-sized" NGOs (MNGOs) and their activities are funded by INGOs or other external donor organizations. Some MNGOs provide services directly to community members at this level, while others provide services through small NGOs, FBOs, and CBOs that operate at the local/district levels [17].

### Contribution of the partners

One participant from a small FBO in the Middle zone described their mutual roles and contributions to the collaboration as follows:

*"Currently, we are trying to minimize infection or eradicate it, if we cannot eradicate it, educate those who are infected to go for drugs that will help them to live longer…we are also educating them to reduce stigma…and to get everybody to know that AIDS is real!"*

In their role as current or previous collaborators of the GAC, CSOs undertake several interventions to reach the general population and "Most-At-Risk Populations" (MARPs): identified as female sex workers, men having sex with men, prisoners, and people who inject drugs. These include community outreach (one-on-one, peer education, small/large group discussions), community mobilization for HTC, condom promotion and distribution, film shows and community drama, distribution of information, education and communication materials, and psychosocial support for PLHIV, as well as home-based care and behavior change counseling [17].

Moreover, many participants viewed periodic review meetings organized by the GAC in which implementers do peer reviews of each other’s performance as an important platform for information exchange that helps to build the capacity of CSOs to deliver more effective programming. Additional capacity building initiatives of the GAC include training of implementers on data collection, M&E and peer education techniques and financial reporting and fiduciary issues, using manuals developed by the GAC. Similarly, a large NGO participant from the Northern zone described how the large CSOs help in building the capacity of small CSOs as follows:

*"Because we act as an intermediary between GAC and our partners [CBOs] in the communities, we strengthen that relationship not only by transferring funds to them but we also provide training and pass on skills, not just those that the GAC provides but what we ourselves as an organization have…For instance, they may have problems developing skills in M&E, budgeting, computer applications, and fundraising…the M&E forms they are supposed to fill are very difficult to complete…so we help them…we sometimes join them in their field activities, such as HTC and peer education."*

Additionally, a different participant from a small NGO in the Southern zone described the contribution of small NGOs to the partnership more generally as follows:

*"We actually deliver the results, so every quarter; we are supposed to report on what we have actually done. And then there is a monthly review meeting we attend to give presentations and assess what we have done. They give us targets…Apart from providing them with personnel and the results; we give them information that would help improve the program. I think they rely a lot on feedback from us because they are not working in the communities or in the field as we do."*

Thus, while the CSOs actually delivered the services, the contribution of the GAC to the partnership has been to coordinate funding from outside donors to build the capacity of the CSOs and to train CSOs to deliver care and prevention more effectively.

### Administration of the partnerships

In order to ensure that its programs are effectively and efficiently coordinated the GAC is organized into a "Projects and Technical" division, an M&E unit, and a finance section. Hence, once a contract is signed with a CSO, clear reporting mechanisms with pre-defined performance targets/indicators are used to evaluate progress. Accordingly, one of the participants from a small FBO in the Southern zone described how their performance is tracked by the GAC as follows:

*"We have reporting templates that we use. So, if for example, we are going to do HTC, we have forms that we use to register people’s details…So they are able to track what we do. They give us the targets. For example, in a year we are supposed to distribute about 200,000 condoms. So you can decide to do the distribution over four quarters…And then at the end of each quarter you show how many you have been able to distribute. That will be your results as against the target set…We do thorough reviews, and at these review meetings, every SR, presents their results and everybody sees it."*

In disbursing funds to the CSOs, a format is usually applied to determine how much they should maintain for themselves, supervision and various budget lines. The results of their implementation activities are collated into a database and used to generate half-yearly and annual reports for dissemination to key stakeholders, including external donors in order to evaluate progress on goals and objectives. Thus, one of the participants from a small NGO in the Southern zone described how the partnership was managed more generally in the following way:

*"The contract basically spells out what we are supposed to be doing, how much we are supposed to be getting, what you do to get your contract taken off us as a SR…So I can say that it is managed by both parties. For instance, we hold review meetings during the implementation stages; they come around on supervisory visits…And during our review meetings we have a session where there is actual feedback…we see whether there are any gaps, whether there are problems, whether there is the need to change anything…"*

To sum up, the GAC has well-defined administrative structures, procedures and reporting requirements for evaluating the performance of CSOs.

### Successes of the partnerships

Concerning the wider successes of the GAC-CSO partnerships, one of the GAC participants underscored how CSOs had over time become good performers in helping to bring the HIV/AIDS epidemic under control through the capacity-building programs of the GAC and funding from external donors. Similarly, one small CSO participant from the Middle zone expressed how beneficial the partnership has been to CSOs and the credibility the relationship enables them to procure:

*"By joining this relationship, we have not only gotten funding but also a lot of information, knowledge, materials, M&E skills that have helped to build our capacity to design and implement high quality HIV/AIDS-related activities…Besides, because we work with the GAC as a state agency, it raises the profile and credibility of our organization such that most donors feel safe funding us."*

Moreover, most of the CSOs suggested that by working together they have been able to reach larger segments of the population with HIV treatment and prevention messages than if they were working independently. Regarding this, one of the participants from a small CBO in the Southern zone explained:

*"Without the funding from the GAC, we would not have been able scale-up our HTC services…we used to do that on a small scale and few people knew their status. But now we have close working relationships with the district assemblies, and the health facilities are more comfortable working with us…So for me HTC is one aspect that we have made achievements…and also condom distribution. For instance, we bought about 700 packets of condoms, but currently we have only a few left…the patronage is encouraging. So it means we are making an impact."*

Thus, external donor funding made available through the GAC, opportunities for building the capacity of CSOs and their distinctive strengths in mobilizing and inducing action on the part of the communities to positively respond to the national HIV/AIDS epidemic are considered success factors of their relationships.

### Challenges of the partnerships

First, a majority of the CSOs attested to delays in the release of funds and tendency of the GAC to reduce initially agreed upon funds in the contract agreement as one of the critical challenges that impacts their work. Regarding this, a large NGO participant from the Northern zone responded by stating:

*"The fact is, because it’s basically donor funding that we are relying on, once the expected funding does not come in or delay, everything comes to a halt. During the last project they were supposed to, by the terms of the contract give about GH¢80,000…and the first installment of 50 % was paid in line with the agreement…the second installment of 50 %, which is GH¢40,000 was supposed to be paid but it was delayed for over four months and they ended up paying only GH¢20,000 towards the end of the project."*

Concerning this, a different participant from a large NGO in the Southern zone expressed worry about the bureaucracy associated with accessing funds from the GAC as follows:

*"What has been a challenge sometimes when it comes to working with government partners has to do with the bureaucracies in terms of delivery or disbursement of funds to organizations to work with and the hierarchies that we have to go through in accessing funds."*

Second, several of the CSOs also expressed dissatisfaction with the tendency for the GAC to make grants on an annual basis instead of having the grants as multi-year commitments. Annual grants were found to adversely affect not only the sustainability of their programs, but also, their survival as organizations. Instead, the CSOs proposed that, having multi-year grants that last between two and five years will enable them to make long-term commitments to resources and staff and to invest in long-range targeting and planning to more effectively and efficiently respond to the national HIV/AIDS epidemic.

At the same time, participants from the GAC emphasized that their financial support from key development partners, such as the USAID, DANIDA, DFID and Global Fund has been declining in recent years, which is blamed on the current global financial crisis and fears that this could negatively impact their response to the national HIV/AIDS pandemic. They also indicated that increased governmental funding is critical if efforts to date are to be sustained and have a long-lasting positive effect.

Apart from issues of funding, most of the small CSO participants noted the tendency for the GAC to set unrealistic targets for HTC activities and condom distribution, as major obstacles to their performance. For HTC activities, set targets were often hard to meet due to limited supplies of HIV testing kits from the GHS, which due to shortages, often restricted HTC to essential activities, such as testing pregnant women and ensuring blood safety. In other situations, the kits may be available but the stringent bureaucratic processes CSOs have to go through to obtain the kits can be frustrating. As a result, the effort, time and money invested in the peer educators who normally mobilize and counsel community members for HTC are compromised. This tends to undermine the whole idea of helping people to know their HIV status as a key ingredient to encouraging people to make informed choices in the fight against HIV/AIDS. Concerning condom distribution, set targets could often not be met, due to a recent directive from the GAC to sell the condoms, which were being distributed free of charge. This had resulted in huge stocks of the commodity remaining unsold. As a result, the CSOs suggested the need for the GAC to revisit its stance on the sale of condoms to increase access to them as a vital commodity for preventing and/or reducing new HIV infections country-wide.

Several participants also reported instances where reactive cases from HTC programs were not linked to care and treatment by health personnel in certain districts as a major challenge to HIV treatment and prevention efforts, such as highlighted in the following quote from a small NGO in the Southern zone:

*"…the main problem is the referrals, because we go to the schools to do HTC with GHS personnel…we get reactive cases but they keep the records to themselves…so if we don’t follow-up on the cases then nothing gets done. So, most at times, we test the people and they show positive…but they are left alone and not taken care because of a poor referral system."*

Moreover, some of the participants complained about non-participation of CSOs in the negotiation of the terms and conditions of their contract and the tendency for the GAC to simply impose the contract on them as unfair. Most participants indicated that they would prefer to have the terms and conditions of the contract negotiated and agreed upon before the two parties' sign, such as what is reflected in the following statement by a large NGO in the Northern zone:

*"…they [GAC] need to re-look at the partnership agreement and involve us from the onset in defining clearly what our role and theirs is going to be…Because what is happening now is that we do not make any inputs or negotiate on the terms of the contract. They have a standard agreement for everybody to just sign. So we tend to have many challenges before the program ends."*

On another note, one GAC participant from the Southern zone stressed the high staff turnover rate among the CSOs as a serious problem of the collaboration in the following way:

*"We train people and all of a sudden they are gone and we have to train new people again…these trainings cost money, so if you have to be repeating this over and over again… the ability to gain and keep the experience becomes a problem and our coordination efforts suffer…"*

Thus, funding issues and other structural barriers constitute key influences on the adverse performance of the partnerships.

## Discussion

This article has provided an overview of on-going state-civil society partnerships for HIV prevention and treatment in Ghana. Major findings include that the commitment by government and CSOs to work in collaboration with external donors to implement decentralized organizational structures and specific administrative policies and practices allow for more successful performance and sustainable involvement in responding to the HIV/AIDS pandemic in Ghana. In particular, this study attests to how this can be done by implementing guiding principles and modes of operation that result in increased access to resources and improved service provision that no one party working alone would have achieved. This inherently increases the efficiency and effectiveness of state-civil society partnerships overall, and implies that the original goals of the GAC to embark upon CSOs’ involvement in the national HIV/AIDS response are being met.

The involvement of CSOs has been found to be valuable in many ways. These include

their role in: 1) delivering more cost-effective HIV/AIDS services through their community mobilization, capacity building, information, resources and skills exchange; 2) complementing and extending the delivery of public sector services, including the provision of more comprehensive HIV/AIDS services that suit local needs and challenges; 3) increasing community mobilization for the HIV/AIDS response, with improved M&E and accountability through the adoption of results-based mechanisms-essential for proper coverage and use of allocated resources; 4) promoting positive behavior change and enhanced prevention through community outreach (one-on-one, peer education, small/large group discussions), use of film shows and community drama; 5) improving communication networks through international, national, regional and local/district CSO linkages, capacity building, information exchange and sharing lessons for more effective HIV/AIDS programming; and 6) promoting representation of the perspectives of CSOs (NGO networks and coalitions), including those of vulnerable groups and underserved communities (e.g., MARPs, PLHIV) in national HIV/AIDS policies and programs.

With the increasing involvement of CSOs by the GAC, new organizational arrangements are emerging to execute the national HIV/AIDS response. However, the early history of these partnerships prevents this study from drawing any final conclusions on the impact of CSOs’ involvement in the national response to the HIV/AIDS epidemic. Nonetheless, it is possible to delineate at least one major model of the GAC’s successful engagement of CSOs that can be replicated in other settings. This model is one in which the GAC operating with donations disburses funds to few CSOs with larger absorptive capacity who act as intermediaries distributing funds to a larger number of smaller, less equipped NGOs, FBOs and CBOs to execute the country’s HIV/AIDS strategy at the national, regional, district and community levels. Investing and promoting the adoption of this model in other settings might be useful as the search for the most appropriate methods for combating the global HIV/AIDS pandemic continues.

This study also draws attention to significant challenges to effective partnerships, which can be seen as structural: they are linked to the ways in which the GAC and CSOs manage and organize their affairs in the collaboration, and to current trends affecting the national HIV/AIDS response. These include, high NGO staff turnover rate; shortage of HIV testing kits, unrealistic targets set for HTC and condom distribution; the non-participation of CSOs in the negotiation of the terms and conditions of their contractual agreements, failure to link reactive HIV cases to care and treatment; dwindling external donor funding; short funding duration; delays and problems associated with the aid chain: how funding conditions (i.e. documentation requirements and bureaucratic procedures) shape the movement of aid through the GAC to CSOs for work in the national HIV/AIDS response. Arguably, most of these obstacles can be addressed through targeted new polices, mechanisms and tools, moving forward to more effectively and efficiently engage CSOs in addressing the HIV/AIDS epidemic.

### Limitations

This study has limitations. Namely, the study was conducted in only one developing country – Ghana. Future research should increase the scope of the study in other developing countries to provide a more comprehensive picture of on-going state-civil society relations in response to the global HIV/AIDS pandemic in order to establish the universality of this study's findings for replication in other settings. This study underscores successes and challenges to date that are associated with state-civil society relations in the fight against the national HIV/AIDS epidemic; longitudinal research is needed to study the influence of suggested changes on state-civil society partnerships in Ghana.

## Conclusions

Based upon the findings of this study, conclusions include that attention needs to be paid to the major barriers to funding identified by the GAC and CSOs such as, scarcity and reductions in allotted money, delays in disbursement, and short funding duration for more successful performance of the partnerships. Incidentally, both the GAC and CSOs were in agreement that realizing the full benefits of these partnerships is not simply a matter of providing funding and setting up organizational systems supporting these partnerships. External donors and government in close consultation with CSOs have an important role to play in altering the environment of the "aid chain”: complex application processes and administrative and reporting requirements that shape the free flow of funds to local CSOs and to put in place new rules, procedures and strategies that overcome these barriers. Finally, the GAC and CSOs recognize that the channeling of multilateral, bilateral donor funding through state institutions established to execute national HIV/AIDS responses to local CSOs in developing countries remain a critical challenge. Hence, they recommend sharing of information and best practices on how to successfully engage local NGOs among external donors, governments, CSOs and other HIV/AIDS implementers, as a means of overcoming the challenge.

## Abbreviations

CBOs, Community-based organizations; CSOs, Civil society organizations; DANIDA, Danish international development agency; DFID, Department for international development of the United Kingdom; FBOs, Faith-based organizations; GAC, Ghana AIDS commission; GHS, Ghana health service; Global Fund, Global fund to fight AIDS, tuberculosis and malaria; HIV/AIDS, Human immunodeficiency virus/acquired immune deficiency syndrome; HTC, HIV testing and counseling; INGOs, International NGOs; M&E, Monitoring and evaluation; MARPs, Most-at- risk populations; MNGOs, medium-sized NGOs; NAP+, Ghana network of persons living with HIV/AIDS; NGOs, Non-governmental organizations; PLHIV, People living with HIV; SR, Sub-recipients; USAID, United States agency for international development

## Additional file

Additional file 1: Interview guide: Interview Guide for Case Study Partnerships’ Representatives. (DOC 48 kb)
